# Transcriptomic Analysis of Radish (*Raphanus sativus* L.) Spontaneous Tumor

**DOI:** 10.3390/plants10050919

**Published:** 2021-05-03

**Authors:** Alexander Tkachenko, Irina Dodueva, Varvara Tvorogova, Alexander Predeus, Olga Pravdina, Ksenia Kuznetsova, Ludmila Lutova

**Affiliations:** 1 Department of Genetics and Biotechnology, Saint-Petersburg State University, 7/9 Universitetskaya Emb., 199034 St Petersburg, Russia; castorfiber@list.ru (A.T.); krubaza@mail.ru (V.T.); pravdina22@gmail.com (O.P.); kskuz95@mail.ru (K.K.); la.lutova@gmail.com (L.L.); 2 Laboratory of Computer Technologies, ITMO University, 197101 St. Petersburg, Russia; 3 Bioinformatic Institute, 194100 St. Petersburg, Russia; predeus@gmail.com

**Keywords:** *Raphanus sativus*, spontaneous tumor, RNA-seq, differential gene expression, cell division, stress response

## Abstract

Spontaneous tumors can develop in different organs of various plant species without any pathogen infection and, as a rule, appear in plants with a certain genotype: Mutants, interspecific hybrids, etc. In particular, among the inbred lines of radish (*Raphanus sativus* L.), lines that form spontaneous tumors on the taproot during the flowering period were obtained many years ago. In this work, we analyzed the differential gene expression in the spontaneous tumors of radish versus the lateral roots using the RNA-seq method. Data were obtained indicating the increased expression of genes associated with cell division and growth (especially genes that regulate G2-M transition and cytokinesis) in the spontaneous tumor. Among genes downregulated in the tumor tissue, genes participating in the response to stress and wounding, mainly involved in the biosynthesis of jasmonic acid and glucosinolates, were enriched. Our data will help elucidate the mechanisms of spontaneous tumor development in higher plants.

## 1. Introduction

The tumor (syn.: Neoplasm) is a pathological structure emerging as a result of uncontrolled proliferation of a group of cells leaving the systemic control of growth rate, cell differentiation, and proliferation. Therefore, the elucidation of the mechanisms of tumor formation may help identify the key regulators of systemic mechanisms controlling cell proliferation and differentiation. Tumor-like structures are found in almost all multicellular organisms, including higher plants. Pathogen-induced tumors, which make up the majority of neoplasms in higher plants, develop under the influence of infectious agents (bacteria, viruses, fungi, nematodes, insects, etc.), which create a niche for their own habitation in the host plant’s organism mostly by shifting the phytohormonal balance and sometimes activating the meristematic competence of plant cells or modulation of the plant cell cycle. At the same time, much rarer spontaneous tumors of higher plants are formed in plants with certain genotypes (mutants, interspecific hybrids, inbred lines) in the absence of any pathogen, which make them closer to animal tumors [1].

**Figure 1:**
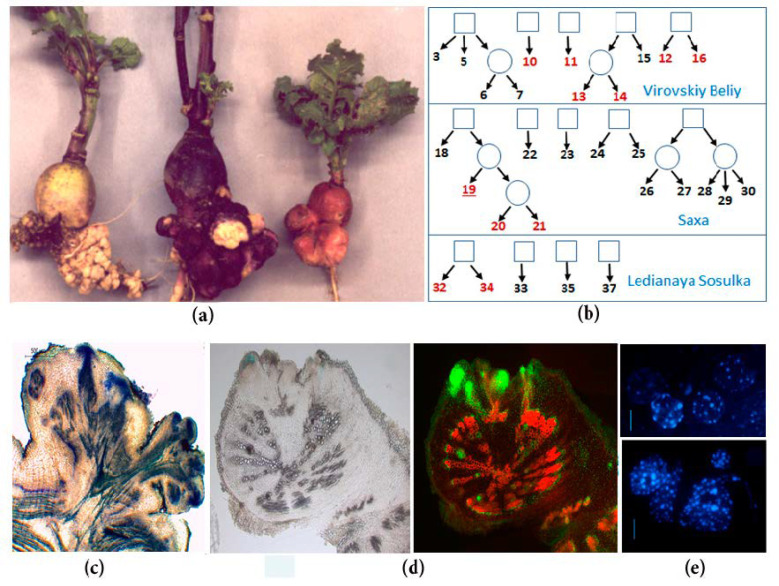
Spontaneous tumors of radish inbred lines: (**a**) Spontaneous tumors on the taproots of different inbred lines of SPbU genetic collection of *Raphanus sativus* (from left to right: Lines 34, 16, and 19)*;* (**b**) the origin of inbred radish lines of SPbU genetic collection. Lines with spontaneous tumor formation are marked in red; (**c**) anatomy of mature spontaneous tumors in radish: Mass of undifferentiated cells in the periphery of tumor, vascularization of the proximal part of tumor, and the connection of tumor and plant vascular systems; (**d**) analysis of cell proliferation intensity in the radish taproot with young tumors: Cells with active DNA synthesis were incorporated EdU (5-ethynyl-2’-deoxyuridine) and fluorescently labeled with Alexa Fluor-488 (right) [5]; (**e**) cytological analysis of roots and tumors in radish inbred line 19 (DAPI staining): Tumor cells (below) have an increased number of chromocenters, which indicate an increased level of ploidy in comparison with taproot cells (above) [7].

The object of our research is spontaneous tumorigenesis in inbred lines of radish (*Raphanus sativus* var. radicula Pers.). The genetic collection of radish was created in St. Petersburg State University by selfing individual plants of four cultivars [2], and now it contains 32 highly inbred lines [3]. Eleven radish lines of different origins are characterized by the spontaneous formation of tumors on the roots of plants at the beginning of the flowering stage [3] (Figure 1). Spontaneous tumors in radish originate from the pericycle and cambium cells [4,5], which brings them closer to the lateral and adventitious roots. Moreover, young growing tumors contain meristematic foci in the periphery, which resemble root apical meristems due to the presence of indole-3-acetic acid (IAA) response maxima and *RsWOX5* expression [5]. The genetic analysis revealed that the spontaneous tumor formation in radish is a polygenic trait, however, it is also inherited as a monogenic recessive trait in several hybrid combinations [6]. As with most examples of spontaneous tumors in plants, the exact cause of spontaneous tumor formation in radish inbred lines is unknown, but the most likely reason is associated with an increase of free cytokinins (CKs) content, which was found in the roots of tumor-producing radish lines [6].

In the present study, we used RNA-seq to analyze the changes in gene expression at the early stages of development of spontaneous tumors in radish inbred line 19, while the lateral roots of the same plants were used as the control. The data we obtained revealed the differential expression of more than 1600 genes. We performed the analysis of GO, KEGG, and GSEA categories and also individual transcripts and revealed the upregulation of genes of pathways associated with cell division and expansion, while among the downregulated, the genes involved in stress response were overrepresented. Our findings were confirmed with qPCR for selected transcripts. Since the examples of spontaneous tumors in plants are very rare, and the mechanisms of their development are poorly understood, our study contributes to the understanding of the genetic control of spontaneous tumors development in radish inbred lines in particular and in higher plants in general.

## 2. Results

### 2.1. RNA-seq of Radish Spontaneous Tumors

Spontaneous tumors of radish, such as lateral roots, are of pericyclic origin and, possibly, represent some modification of the lateral root development program [5]. To compare transcriptomes of spontaneous tumors and lateral roots of radish, we isolated the RNA from tumors and lateral roots of radish plants of tumor-producing line 19 and subjected it to sequencing. Total RNA from three replicates of root and tumor samples was sequenced with an Illumina HiSeq2500 sequencer resulting in 190.3 million paired-end reads. After all adapter trimming and contamination removal steps, 73.8 million paired-end reads were used for differential expression estimation and all downstream analyses.

**Figure 2:**
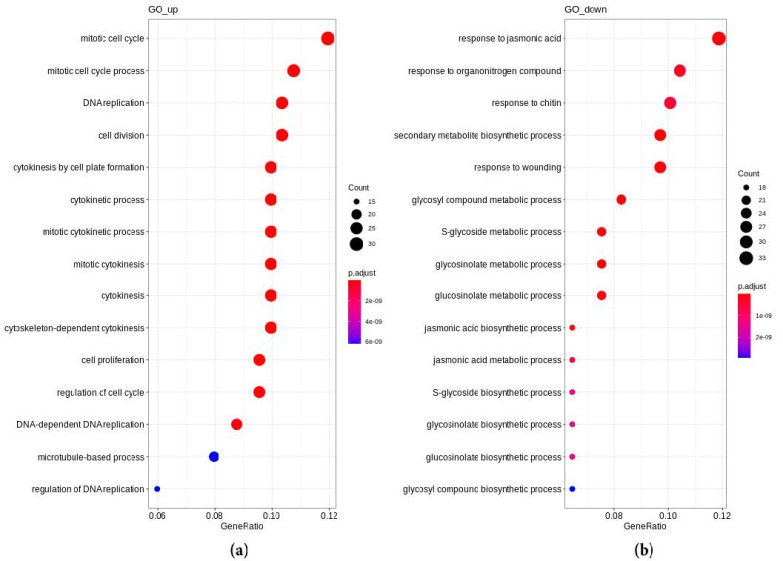
Overrepresented “biological process” GO pathways in genes upregulated (**a**) and downregulated (**b**) in the spontaneous tumors of radish in comparison with lateral roots. The count is the number of DEGs included in the respective pathway.

We have found that 425 genes were significantly upregulated in young tumors compared to lateral roots, while 1203 genes were significantly downregulated (adjusted *p*-value < 0.05). Data on differential expression of genes were further analyzed using the gene set enrichment analysis (GSEA) method with the fgsea package. Three hundred and forty-four groups of genes (out of 9831 groups taken for the analysis) were significantly enriched among the genes with increased expression in radish tumor samples, and 132 groups of genes were significantly enriched among the genes with reduced expression in the tumor compared to the lateral root (adjusted *p*-value < 0.05) (Table S2). The gene ontology enrichment analysis performed with the clusterProfiler package on significantly differentially expressed genes has shown that 76 biological process terms were enriched among upregulated genes and 99 were enriched among downregulated genes with adjusted *p*-value < 0.05 (Figure 2, Table S2), whereas the KEGG enrichment analysis has shown that 8 and 15 pathways were enriched among upregulated and downregulated genes, respectively (adjusted *p*-value < 0.05) (Table S2).

### 2.2. Enriched Pathways in Genes Differentially Expressed between Spontaneous Tumors and Lateral Roots

**Figure 3:**
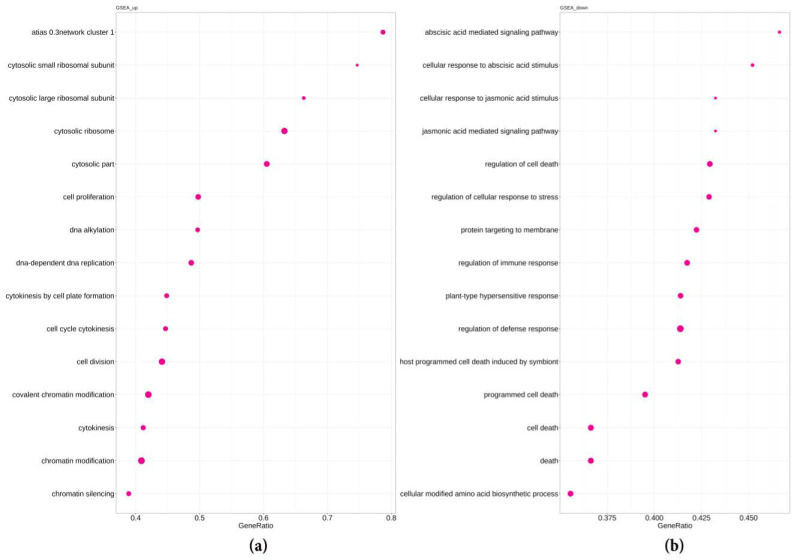
Overrepresented “biological process” GSEA pathways in genes upregulated (**a**) and downregulated (**b**) in the spontaneous tumors of radish in comparison with lateral roots. The count is the number of DEGs included in the respective pathway, the adjusted *p*-value is 3.4 × 10^−9^.

We analyzed GO, GSEA, and KEGG pathways in lateral roots and spontaneous tumors of radish line 19. The data are presented in Table S2 and in Figure 2 and Figure 3.

According to the GO pathways analysis, among the upregulated in young spontaneous tumors (Figure 2a), the most enriched were pathways associated with the regulation of hyperplasia: Cell cycle (GO:1903047, GO:0000278, GO:0008283, GO:0051301, GO:0051301, GO:0051726, GO:0010389, GO:1902749, GO:0000086, GO:1901987, GO:1901990, GO:0044770, GO:0044772, GO:0007346, GO:0010564, GO:0045786), DNA replication (GO:0006260, GO:0006261, GO:0006275, GO:0006270, GO:0042023, GO:0044786), cytokinesis (GO:0000911, GO:0032506, GO:1902410, GO:0000281, GO:0000910, GO:0061640), spindle assembly (GO:0051225, GO:0007051), cytoskeleton rearrangement (GO:0007017, GO:0000226, GO:0007010), cell expansion (GO:0009825), and cell wall modifications (GO:0009828, GO:0042547, GO:0009831, GO:0009505), which reflect the activation of cell division and growth in the developing tumor in comparison with the lateral root. The upregulation was noted for pathways involved in chromatin modification (GO:0016570, GO:0034968, GO:0016569, GO:0016571, GO:0016572, GO:0051567, GO:0061647, GO:0006305, GO:0006306, GO:0044728, GO:0006304, GO:0000785) and gene silencing (GO:0016458), response to hormones (CK—GO:0009736, GO:0071368, and gibberellic acid (GA)—GO:0009739), numerous pathways associated with amino acid and protein modifications (GO:0018022, GO:0006479, GO:0008213, GO:0018205, GO:0018193), DNA metabolism (GO:0051052), organelle development (GO:0070925, GO:0031225, GO:0022626, GO:0044445), and morphogenetic processes (GO:0010374, GO:0048646, GO:0048831).

At the same time, among downregulated (Figure 2b) genes, there were numerous stress-related pathways such as response to wounding (GO:0009611), response to abiotic stress (GO:0080135, GO:0006979, GO:0010193), and immune response (GO:0045088, GO:0050776, GO:0002682, GO:0050832, GO:0009625, GO:0031348, GO:0010200), in particular, of hypersensitive response (HR) GO:0010363, GO:0009626) and systemic acquired resistance (SAR) (GO:0009862) reactions, and cell death (GO:0043067, GO:0010941, GO:0012501, GO:0034050, GO:0008219). Among downregulated genes, there were also pathways of biosynthesis, metabolism, and signaling of different phytohormones, which can also participate in plant stress response: jasmonic acid (JA) (GO:0009753, GO:0009695, GO:0009694, GO:0009867, GO:0071395), salycilic acid (SA) (GO:0009751, GO:0009863, GO:0071446), ethylene (GO:0009692, GO:0009693, GO:0009723), IAA (GO:0009684, GO:0009683, GO:0042435, GO:0009851, GO:0009850, GO:0009733, GO:0042436), and even karrikins (GO:0080167). The activation of some metabolic pathways, such as biosynthesis and metabolism of ROS compounds (GO:0010310, GO:2000377, GO:0016702, GO:0016701), phenylpropanoids (GO:0009698, GO:0009699), flavonoids (GO:0009812, GO:0009813, GO:0009963, GO:0009962), glucosinolates (GO:0019757, GO:0019760, GO:0019758, GO:0019761), and polyamines (GO:0006595, GO:0006598), may also be associated with the response to stress. The downregulation was also found for pathways associated with amino acid metabolism and transport (GO:0019344, GO:0006534, GO:0009070, GO:0009069, GO:0003333, GO:0006865), and also the transport of organic acids (GO:0015849, GO:0046942) and anions (GO:0015711, GO:0015698).

The data obtained in the analysis of the GSEA and KEGG pathways (Table S2), in general confirm the data obtained for the GO pathways. For instance, among upregulated GSEA pathways in spontaneous tumors (Figure 3, Table S2), those associated with cell division and chromatin modification were presented. Moreover, among the enriched, there were GSEA pathways associated with the ribosome biogenesis and also the development of meristems and floral organs. At the same time, among downregulated GSEA pathways in the spontaneous tumors, the pathways associated with the response to JA, ABA, as well as stress and immune responses were prevailed.

Among the most enriched KEGG pathways in the tumor tissue, there were also those associated with cell division (e.g., DNA replication and mismatch repair), and among the downregulated, the most enriched were the pathways of biosynthesis of glucosinolates and phenylpropanoides. Thus, according to the data on enriched pathways determined by different classifications (GO, GSEA, and KEGG pathways analyses), most of the upregulated pathways in spontaneous tumors were related with cell division and chromatin modification, and most of the downregulated pathways were associated with stress response.

### 2.3. Individual Transcripts Differentially Expressed between Spontaneous Tumors and Lateral Roots

In our experiment, numerous genes that are differentially expressed were identified between the spontaneous tumor and the lateral root: 425 genes were significantly upregulated and 1203 genes were downregulated in the spontaneous tumors of radish (Table S2).

As noted above, the large number of genes upregulated in spontaneous tumors control cell division and elongation, while genes involved in the stress response were widely represented among the downregulated ones. The exact functions of these genes in radish are unknown, however, the functions of their closest homologues in *Arabidopsis* were previously studied. Due to the high conservatism of the pathways of cell cycle control [8], as well as JA biosynthesis and stress response [9] in higher plants, we can assume that the radish DEGs identified by us probably perform functions similar to those of their homologues in *Arabidopsis*.

#### 2.3.1. Cell Division and Cell Expansion Genes: Mostly Upregulated

**Figure 4:**
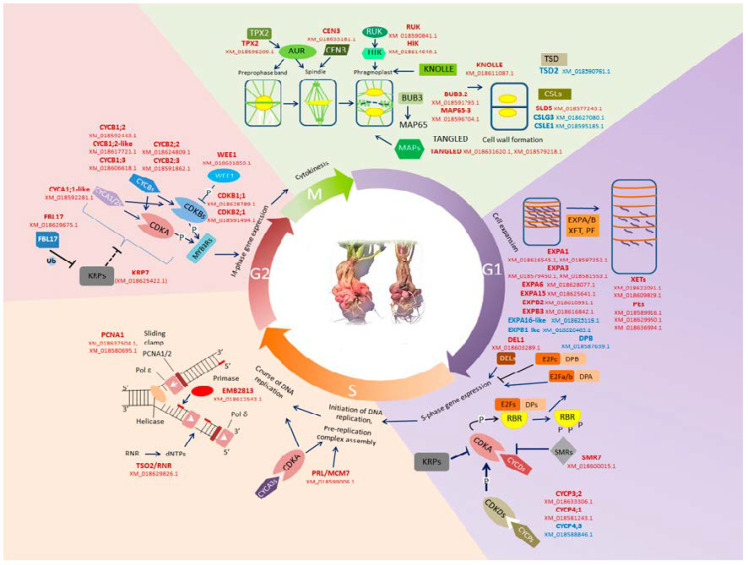
Scheme of genetic control of the plant cell cycle; genes whose homologues were differentially expressed in spontaneous tumors of radish are marked in color (red—upregulated genes, blue—downregulated genes). See the text for an explanation.

As it was shown in anatomical studies, the development of tumors of various types in plants is associated with the intensification of cell division and cell elongation [1,10]. According to this, a lot of genes associated with mitotic cell cycle and most of the genes associated with cell growth were upregulated in spontaneous tumors (Figure 4) reflecting the increased level of cell proliferation during the formation of neoplasm.

As in the analysis of pathways, among the individual upregulated transcripts, there were radish homologs of Arabidopsis genes that are involved in the control of different stages of the cell cycle and cytokinesis: Genes encoding cyclins of different classes, cyclin-dependent kinase B (CDKB), E2F and DP transcription factors, WEE1 kinase, cell cycle inhibitors of KRP and SMR families, proteins involved in the DNA replication and in the cytoskeleton dynamics during mitosis, and also genes, encoding cell wall loosening enzymes, which are essential for cell expansion (Figure 4). An increase in the expression levels of these genes in the spontaneous tumors may be the cause for the intensification of cell divisions in the tumor tissue.

At the G1 phase of plant cell cycle, the growth of plant cell takes place requiring cell wall destabilization, which depends on the activity of cell wall modifying enzymes such as expansins (EXP), pectinesterases (PE), and xyloglucan endotransglucosylases/hydrolases (XET) [11]. Radish homologs of *Arabidopsis EXPA1, EXPA3*, *EXPA6, EXPA15, EXPB2, EXPB3,* along with three *PE* genes and two *XET* genes were upregulated in the spontaneous tumors, and only two *EXP* genes were downregulated. In addition, *TUMOROUS SHOOT DEVELOPMENT 2/QUASIMODO 2* (*TSD2/QUA2*) encoding pectin methyltranspherase, which is essential for cell adhesion was downregulated in the tumor tissue [12].

The G1-S transition of plant cell cycle is under the control of A-class cyclin-dependent kinases (CDKA) and D-class cyclins (CYCDs) [13,14]: CDKA-CYCDs phosphorylate the retinoblastoma-related (RBR) protein and release transcription factors (TFs) of E2F/DP family from an interaction with RBR [13,14]. The E2F and DP proteins form heterodimers, while DP-E2F-like (DEL) proteins do not dimerize [15]. The E2F-DP and DEL TFs regulate the expression of S phase genes [16,17]. Among DEGs in spontaneous tumors, there were radish homologs of *Arabidopsis E2FE/DEL1* and *DPB* genes: homolog of *DEL1* was upregulated in the tumors, while homolog of *DPB* was strongly downregulated.

The main event of S phase of the plant cell cycle, the course of which is under the control of CDKA-CYCA3 complexes [18], is DNA replication. In the spontaneous tumors of radish, several genes encoding important regulators of DNA replication were upregulated. Among them were radish homologs of *PROLIFERA* (*PRL),* which encode a key component of the pre-replication complex [19], *PROLIFERATING CELLULAR NUCLEAR ANTIGEN 1* (*PCNA1*) whose product forms the DNA clamp [20], *TSO2* whose product catalyzes the synthesis of deoxyribonucleosides [21], *EMBRYO DEFECTIVE 2813* (*EMB2813)* encoding a large subunit of DNA primase [22], and *RPA32B* (*AT3G02920*) which encodes one of three subunits of replication protein A (RPA)—an ssDNA-binding protein [23].

The G2-M transition of plant cell cycle is under the control of CDKAs and CDKBs, which interact with B-class and certain A-class cyclins [24], and the main targets of CDKs at G2-M are MYB3R TFs, which regulate the expression of M phase genes [25]. In the spontaneous tumors, the upregulation of genes homologous to *Arabidopsis CDKB1;1, CDKB2;1, CYCA1;1-like, CYCB1;2*, *CYCB1;2-like*, *CYCB1;3*, and *CYCB2;2* was noticed.

The activity of CDK-cyclin complexes at all phases of the plant cell cycle depends on other proteins: CDKDs, activators of CDK, which interact with CYCPs acting in the nutritional control of cell cycle [26,27], and inhibitors of CDK—*SIAMESE-RELATED* (SMR) [28] and Kip-related proteins (KRPs) [29]. In turn, the stability of KRPs depends on the F-box protein FBL17 [30]. At G2-M, the CDK activity is also under the control of the WEE1 kinase, which phosphorylates and inhibits CDKs [31]. Among genes which were upregulated in the spontaneous tumors of radish, there were homologs of *Arabidopsis CYCP3;2, CYCP4;1, SMR7, KRP7, FBL17,* and *WEE1,* while among downregulated genes, there was a homolog of *CYCP4;3*.

At the M phase of cell cycle, various regulators of spindle formation and cytokinesis play a key role. Radish homologs of several genes from these groups were upregulated in the spontaneous tumors, including genes encoding microtubule-associated proteins *TANGLED* and *MAP65-3/PLEIADE,* which play a role at early and late mitosis [32,33], *BUDDING UNINHIBITED BY BENZYMIDAZOL3.2* (*BUB3.2*), which encodes the mitotic checkpoint protein regulating the localization of MAP65-3 in the phragmoplast [34], *TARGETING PROTEIN FOR XKLP2* (*TPX2*) whose product is an upstream regulator of Aurora A kinase and a participant of mitotic spindle assembly [35], *CENTROMERIC HISTONE H3* (*CEN3*) encoding an assembly site for the kinetochore complex [36], *KNOLLE* encoding cytokinesis-specific syntaxin [37], *RUNKEL* (*RUK)* encoding microtubule-associated kinase [38] together with the gene of RUK target HINKEL (HIK), a kinesin required for phragmoplast expansion [39], and also *SLD5* encoding cellulose synthase, which functions in cell plate formation [40].

It was previously reported that the homologs of cell division and cell expansion genes, which were upregulated in the radish spontaneous tumors can participate in the control of meristem activity and organ size, and also in the development of other types of plant tumors. For example, *CDKB2;1* whose radish homolog is strongly upregulated in the spontaneous tumors is involved in cell cycle progression and determines the meristem size [41]. The protein kinase WEE1 which represses the cell cycle progress by inactivating CDKs dose-dependently decreases the meristem size and regenerative capacity in *Arabidopsis* [42], but the expression of *Arabidopsis* *WEE1* is associated with actively proliferating cells [31]. Therefore, its upregulation in the tumor tissue may be a part of the negative feedback regulation of cells proliferation. It was also reported that the increase of the expression levels of cell cycle and cell expansion genes, such as *DEL1*, *WEE1*, *KRP7*, and certain *EXPAs* and *EXPBs* takes place during the development of galls induced by nematodes [43,44,45,46]. In our previous work, the same radish homolog of *EXPA1* was revealed as one of the most upregulated genes in the transcriptome of *Agrobacterium*-induced crown gall tumor [47]. The list of downregulated genes in the spontaneous tumor includes the radish homolog of *TSD2/QUA2,* while the *tsd2/qua2* mutant belongs to a specific small group of monogenic spontaneous tumor-producing mutants of *Arabidopsis* [1,12]. Therefore, downregulation of *TSD2* homolog may be one of the causes of spontaneous tumor formation in radish.

#### 2.3.2. Stress Response Genes: Mostly Downregulated

**Figure 5:**
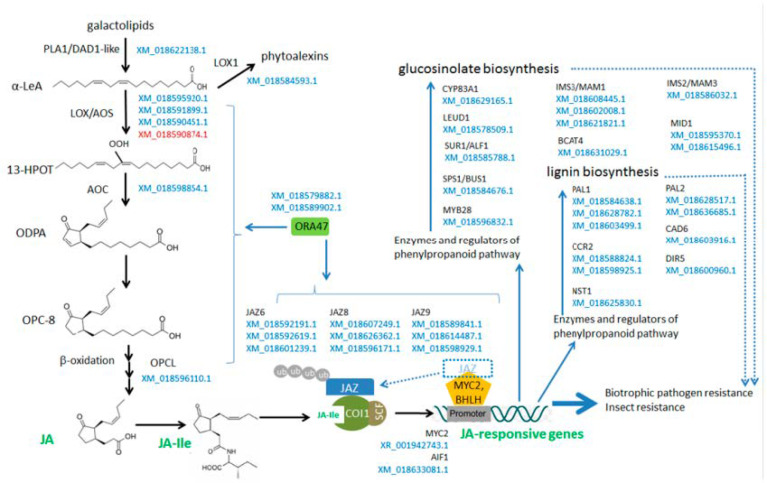
Scheme of several pathways of the plant stress response; genes whose homologues were differentially expressed in spontaneous tumors of radish are marked in color (red—upregulated genes, blue—downregulated genes). See the text for an explanation.

Among the downregulated in the spontaneous tumors, the stress-related genes, namely genes involved in the biotic stress and wounding response, are widely represented (Figure 5).

A significant part of these genes is involved in the biosynthesis and signal transduction of JA, an oxylipin phytohormone involved in the response to biotic and abiotic stress, wounding, pathogenesis, and herbivorous insects [9]. The JA biosynthesis is initiated in chloroplasts by the release of α-linolenic acid (α-LeA) from galactolipids of chloroplast membranes by phospholipase A1 (PLA1)/ DEFECTIVE IN ANTHER DEHISCENCE 1 (DAD1) [48]. The second step of JA biosynthesis is catalyzed by lipoxigenases (LOX)—enzymes which oxidize the α-linolenic acid of chloroplast membranes to the (13S)-hydroperoxyoctadecatrienoic acid (13-HPOT). Among LOX family enzymes, only the allene oxide synthase (AOS) branch, which includes LOX2, LOX3, LOX4, and LOX6, is involved in the JA biosynthesis [49]. In the chloroplasts, AOS enzymes work in tight communication with allene oxide cyclases (AOCs), which cyclize the unstable product of α-linolenic acid oxidation to obtain the 12-oxo-phytodienoic acid (OPDA). The third step of JA biosynthesis is the conversion of OPDA in the peroxisome to 3-oxo-2-(2′-[Z]-pentenyl)cyclopentane-1-octanoic acid (OPC-8), which subsequently undergoes three rounds of beta-oxidation to yield JA [50].

The ubiquitin-dependent degradation of JA-zim-domain (JAZ) proteins plays a central role in the response to JA. These proteins are transcriptional repressors which bind and inhibit the TF MYC2 to repress the JA response, and as a negative feedback in the JA response, JAZ transcript levels rise in response to a JA stimulus [51].

Among genes which were downregulated in radish spontaneous tumors, there were homologs of genes which regulate key steps of JA biosynthesis: DAD1-like Lipase 3 (*DALL3*), *LOX3, LOX4, LOX6,* and *AOC3,* as well as homologs of *OPCL1* gene encoding enzyme, which activate JA biosynthetic precursors in the peroxisomes [50] and *CYP94B3* which encodes a key enzyme in the oxidative catabolism of JA [52]. Among downregulated genes, there were also upstream regulators of JA biosynthesis such as the gene encoding octadecanoid-responsive AP2/ERF-domain TF ORA47 [53]. Among downregulated in the spontaneous tumors, there were also homologs of genes encoding TFs involved in the response to JA *MYC2* and *AIF1,* as well as JA-responsive genes of transcriptional repressors *JAZ6*, *JAZ8*, and *JAZ9* [51,54].

Moreover, among genes which were downregulated in the spontaneous tumor, there were many stress-responsive genes acting probably independently of JA. These genes encode quite different proteins, and some of them are involved in the metabolism, transport or signaling of other stress-related hormones such as ABA, SA, ethylene, as well as genes involved in the response to drought, salt stress, and also local and systemic immune response.

The targets of the stress response in a plant cell can be genes encoding proteins with protective functions and enzymes responsible for the synthesis of antimicrobial and protective substances, as well as detoxification of toxic metabolites [55]. Accordingly, among genes which were downregulated in the spontaneous tumors, there were radish homologs of several genes encoding stress-related enzymes such as metacaspase AMC6 involved in the cell death and hypersensitivity response (HR) to pathogens [56], and GLUTATHIONE-S-TRANSFERASE GSTF11, which play a role in herbicide detoxification and responses to biotic and abiotic stress [57].

Examples of plant metabolites with an antimicrobial or barrier activity that are synthesized in response to stress are lignin, which provides physical and chemical protection for plants against pathogen invasion, and a number of substances that act as natural bacterio-, fungi-, and/or insecticides, such as glucosinolates.

Lignin is a heterogeneous polymer of monolignols, which is polymerized at the surface of the cell walls. It is extremely important for terrestrial plants: lignin provides structural support for the upward growth and also forms a physical barrier to block pathogen invasion, and prevents the ingress or diffusion of toxins from pathogens [58]. Monolignols, structural units of lignin polymers, are synthesized from phenylalanine via the phenylpropanoid pathway. Thus, enzymes of the phenylpropanoid biosynthetic pathway are critical for lignin biosynthesis [59]. In the spontaneous tumors in radish, we revealed the downregulation of radish homologs of genes encoding enzymes, which act at all stages of the phenylpropanoid pathway: genes encoding phenylalanine ammonia-lyases ATPAL1 and ATPAL2, *CCR2* encoding cinnamoyl CoA reductase, gene encoding cinnamyl alcohol dehydrogenase ATCAD6, and also *DIR5,* which encodes the dirigent-like family protein involved in the synthesis of lignans from two molecules of coniferyl alcohol. In addition, among the downregulated, there was a radish homolog of *NAC SECONDARY WALL THICKENING PROMOTING FACTOR 1* (*NST1*) encoding TF, which regulates secondary cell wall thickening via lignification [60].

Among plant antimicrobial substances, glucosinolates are specific for plants of the *Brassicaceae* family [61]. Glucosinolates have direct antimicrobial properties, in addition, upon damage to plants, e.g., by chewing insects, glucosinolates are enzymatically converted into a range of compounds with antimicrobe and insecticide activities, such as isothiocyanates [62]. In the tumor tissue, we revealed the downregulation of radish homologs of genes for glucosinolate biosynthesis enzymes: Cytochrome P450s CYP83A1 and SPS1/BUS1, methylthioalkylmalate synthases IMS3/MAM1 and IMS2/MAM3, isopropylmalate isomerase LEUD1, methionine-oxo-acid transaminase BCAT4, C-S lyase SUR1/ALF1, and 3-isopropylmalate dehydrogenase ATIMD1 [63]. Another downregulated gene, the radish homolog of *MYB28* encoding TF involved in the positive regulation of aliphatic glucosinolate production [64], was presented. It is interesting that in *Arabidopsis*, genes *SUR1*/*ALF1* (*SUPERROOT*/*ABERRANT LATERAL ROOT FORMATION 1*) and *SPS1*/*BUS1* (*SUPERSHOOT*/*BUSHY 1*) encoding enzymes of glucosinolate biosynthesis, are also involved in IAA production and play a role in the development of root and shoot systems [65,66]. At the same time, the situation with a negative regulation of stress-related genes in the spontaneous tumor is not absolute: among upregulated genes, there were also several radish homologs of genes encoding enzymes of JA and SA biosynthesis, such as *AZELAIC ACID INDUCED 1* (*AZI1*), which is involved in the priming of systemic immunity [67], and one of lipoxygenase genes, *LOX2*.

#### 2.3.3. Genes Related with Cytokinins, Auxins, and Gibberellins

CK, IAA, and GA are plant hormones, which play major roles in the control of cell proliferation and expansion, as well as in the meristem development and organogenesis. It is well known that GA and CK exhibit antagonistic effects on various developmental and molecular processes during plant growth and can inhibit signaling of each other [68,69]. For instance, in the SAM and cambium, GA stimulates differentiation of specialized cell types, while CK stimulates stem cells proliferation [70,71]. In lateral root formation, however, both CK and GA inhibit this process influencing the polar transport of IAA [72,73]. IAA is a well-known positive regulator of lateral root formation, which is necessary for the expression of key “root genes” [74], and this process is under the antagonistic control of IAA and CK [75]. Among DEGs which were identified in the spontaneous tumors in radish, there were numerous genes involved in the biosynthesis of IAA, CK, and GA and the response to these hormones.

It is interesting that among upregulated genes in the tumor tissue, there were genes involved in the positive regulation of GA and negative regulation of CK content and signaling, while among downregulated genes, the situation was opposite. For instance, among downregulated genes, there were radish homologs of *IPT5* and *IPT7* genes encoding adenylate isopentenyltransferases, key enzymes of CK biosynthesis [76], and also genes encoding CK-activating enzymes of LONELY GUY family [77]—*LOG1, LOG3-like, LOG4*, and *LOG8*. Among the downregulated, there was also a radish homolog of *ARR1* gene encoding key CK-responsive TF [78].

At the same time, homologs of genes encoding CK dehydrogenases, which are involved in the degradation of active CK [79]—*CKX3* and *CKX3-like*, and also genes encoding repressors of CK signaling [80]—*ARR4* and *ARR5*, were upregulated in the spontaneous tumors. The only downregulated gene encoding CK-inactivating enzyme was the radish homolog of *CKX1*.

The opposite situation was observed for GA: the radish homologs of *KAO2* encoding ent-kaurenoic acid oxidase, enzyme of GA biosynthesis [81], and also gene *GA-20 oxidase-3-like* (probably involved in the production of active GA [82]), were strongly upregulated in the spontaneous tumors. At the same time, among downregulated genes, there was the *GA2OX6* encoding protein of GA 2-oxidase family, which is a key enzyme of GA inactivation [83].

The IAA is the main positive regulator of lateral root development, and the genes involved in the biosynthesis, conjugation, transport, and signaling of IAA were downregulated in the tumor tissue compared to the lateral root. In the list of downregulated genes, there were regulators of polar IAA transport: radish homologs of genes encoding IAA efflux protein PIN7, which establishes an apical-basal axis in the embryo and is involved in the pattern specification during root development [84], as well as AUX1 and LAX3 IAA influx transporters [85]. Among genes involved in the IAA signaling and downregulated in tumors, there were radish homologs of *AUXIN RESPONSE FACTOR 9* (*ARF9)*, encoding IAA-regulated TF, and also *IAA2* and *IAA18* genes encoding transcriptional repressors of Aux/IAA family involved in root development [86,87]. Moreover, among downregulated genes in the spontaneous tumors, there were radish homologs of genes, connecting the IAA biosynthesis with the stress response: *YUCCA8* encoding enzyme of IAA biosynthesis which acts in the JA-dependent regulation of IAA homeostasis [88], acyl acid amido synthetase gene *GH3.5,* whose product conjugates IAA with amino acids but also can conjugate SA to modulate both auxin and pathogen response pathways [89], and *IAR1* encoding IAA-Alanin hydrolase, an enzyme which releases IAA from conjugates with amino acids [90].

#### 2.3.4. Genes Involved in Photomorphogenesis and Flowering

Among DEGs in the spontaneous tumor, there were also several key regulators of circadian clock and photomorphogenesis, and their main targets—regulators of the development of inflorescence meristem and flower organs. Members of this system function in the leaves and SAM, and the identification of their homologs among DEGs between the lateral root and the tumor in the root, was rather unexpected.

First, among downregulated genes, there were radish homologs of CICRCADIAN CLOCK ASSOCIATED (*CCA1*) and *LATE ELONGATED HYPOCOTYL* (*LHY*): in *Arabidopsis*, the transcriptional repressor CCA1 forms a heterodimer with the homeodomain-like superfamily TF LHY and binds to the promoter of *TIMING OF CAB 1* (*TOC1*) gene forming a regulatory feedback loop in the circadian oscillator [91]. The target of this regulatory loop is the *CONSTANS* (*CO*) gene encoding TF with a zinc finger domain [92]. The CO-like (COL) TF family includes about 20 proteins involved in the flowering time control in *Arabidopsis* and radish [93], and *RsCOL5*, a radish homolog of *Arabidopsis CO-like 5* was downregulated in the spontaneous tumor. The main target of TF CO in the leaf is the *FLOWERING LOCUS T* (*FT*) gene, which encodes a short mobile peptide known as florigene [94]. In *Arabidopsis* and other plant species, there are many FT-like peptides each of them playing a specific role in the day length-dependent control of flowering (there are florigenes and antiflorigenes among them), and also in some other processes such as the development of adventitious buds, potato tubers, and bulbs [95]. Among downregulated genes in the spontaneous tumor, there was a radish homolog of *BROTHER OF FT AND TFL1* (*BFT*) gene encoding FT-like protein, which functions as a repressor of flowering [96]. In the shoot apical meristem, FT-like proteins interact with FLOWERING LOCUS D (FD) family TFs, and the targets of this complex are inflorescence meristem identity genes such as the *SUPRESSOR OF OVEREXPRESSION OF CONSTANS* *1* (*SOC1)* and *APETALA1* (*AP1*). In turn, the TFs SOC1 together with AGAMOUS-LIKE 24 (AGL24) positively regulate the expression of floral meristem identity gene *LEAFY* [97]. The radish homologs of key inflorescence meristem identity gene *SOC1* and one of the indirect targets of SOC1 TF in the flower, *PISTILLATA* (*PI)* [97], were downregulated in the spontaneous tumors. Moreover, among genes downregulated in spontaneous tumors, there were homologs of *RADIALIS-like3* and *DIVARICATA* genes, encoding TFs which regulate flower symmetry [98]. The expression of these genes in the radish root and their downregulation in the spontaneous tumor allow us to suppose the broader function of the flowering control system, e.g., in the regulation of root development.

### 2.4. Verification of Differential Expression Analysis Data with qPCR

Next, we performed the qPCR expression analysis for some genes that showed an increase or decrease in their expression levels in the spontaneous tumors of radish according to the transcriptome analysis. In total, we analyzed 12 upregulated and 12 downregulated genes by qPCR. To order to conduct qPCR, we selected genes involved in different pathways and associated with varied functions.

Among upregulated genes, we analyzed the expression of radish homologs of genes involved in the control of cell cycle (*CYCA1;1* (*XM_018592281.1*), *CYCB1;2* (*XM_018617721.1*), *DEL1* (*XM_018603289.1*)) and cell growth (*EXPA3* (*XM_018581553.1*)), biosynthesis of GA (*GA20OX3* (*XM_018633395.1*)), negative regulation of cell response to cytokinin (*ARR4* (*XM_018624193.1*) and *ARR5* (*XM_018581651.1*)), genes encoding peptide phytohormone *CLE46* (*XM_018604748.1*), TFs of different families (*LBD25* (*XM_018597567.1*), *LBD38* (*XM_018581284.1*), *WRKY9* (*XM_018604382.1*)), and also the homolog of unique bifunctional gene *ENO2/MBP1* (*XM_018580867.1*) encoding glycolytic enzyme enolase ENO2 and TF MBP1, which alternatively translated from the same transcript [99]—this gene was in the first place among those upregulated in spontaneous tumors.

Among genes which were downregulated in the tumor tissue, we took into analysis radish homologs of genes involved in the cell cycle regulation (*DPB* (*XM_018587639.1*)), genes encoding enzymes of biosynthesis of JA (*AOC3* (*XM_018598854.1*)), IAA (*YUC8* (*XM_018594055.1*)), CK (*IPT5* (*XM_018587590.1*) and *IPT7* (*XM_018633594.1*)), and glucosinolates (*IMS3* (*XM_018608445.1*)), TF-encoding genes (*TCP2* (*XM_018608972.1*), *RL3* (*XM_018614337.1*), *NAC090* (*XM_018588875.1*), *SOC1* (*XM_018623442.1*), *BEL-like 4* (*XM_018603518.1*)), and also the gene encoding FT-like protein BFT (*XM_018581284.1*).

**Figure 6:**
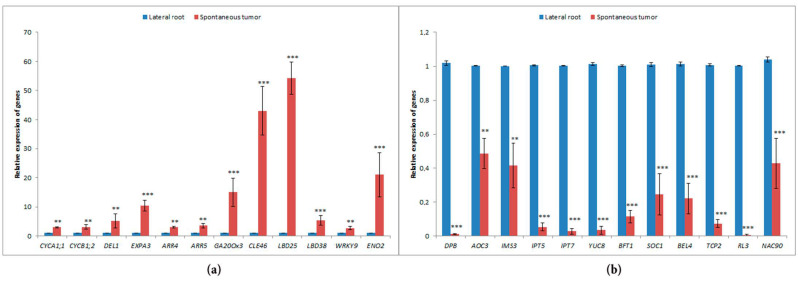
Gene expression analysis by qPCR: (**a**) Genes which were upregulated in the RNA-seq experiment; (**b**) genes which were downregulated in the RNA-seq experiment. Error bars indicate the standard deviation of three technical repeats (*p*-value < 0.01—**, *p*-value < 0.001—***).

In general, the obtained results of qPCR confirmed the transcriptome data: genes that have been identified as upregulated and downregulated in the RNA-seq experiment, demonstrated a significant increase or decrease of expression levels in the qPCR analysis, respectively (Figure 6).

## 3. Discussion

Tumors of higher plants are insufficiently studied, but represent a very interesting phenomenon, since they arise due to the release of a population of cells out of a very reliably arranged systemic control of the proliferation of plant cells. Spontaneous tumors in higher plants are especially rare, and the exact mechanisms of development of most of them remain unclear. In recent years, technologies of next-generation sequencing (NGS) have proven to be instrumental for studying various biological phenomena in different plants [100,101]. RNA sequencing (RNA-seq) is used to assess the functional state of biological tissue based on the variety and abundance of RNA molecules in the sample, so RNA-seq makes it possible to identify candidates for the role of regulators of plant tumor formation. To date, RNA-seq approaches were used for studying the gene expression during the development of only one type of plant tumor, the crown gall caused by *A. tumefaciens* [47,102]. In our work, we used RNA-seq to analyze the changes in gene expression in the spontaneous tumor of the radish inbred line compared with the lateral root of the same line of radish.

We obtained data demonstrating the significant difference between spontaneous tumors and lateral roots in gene expression profiles: both by pathways and by individual transcripts. The data that seemed most interesting to us were also verified by qPCR. In particular, in the spontaneous tumors, we observed a significant activation of genes associated with the control of cell division: among the pathways, the most enriched were those associated with the control of the cell cycle, DNA replication, cytokinesis, and cell growth. Among numerous upregulated genes involved in cell division, the genes controlling the processes of the G2-M transition and cytokinesis were most widely represented. Moreover, among them there were radish homologs of genes encoding the central participants in these processes in *Arabidopsis*, such as genes of CDKBs and its interacting partners, B-class cyclins, which provide the G2-M transition [103], as well as such important regulators of cytokinesis as centromere-associated histone H3 variant CEN3, which represents the assembly site for the kinetochore complex of active centromeres [36] or syntaxin KNOLLE, which is essential for phragmoplast formation [37]. The upregulation of genes involved in the control of the cell cycle and cytokinesis reflects the high frequency of cell divisions in spontaneous tumors (Figure 1). In addition, among the upregulated “cell division genes”, there were also inhibitors of CDKs, an increase in the expression of which is usually associated with an increase in the frequency of endomitoses, e.g., during the formation of one of the types of plant tumors—root galls induced by nematodes [43,44]. Indeed, in the spontaneous radish tumors, a high level of polyploid cells was noted compared to normal root tissues (Figure 1). Finally, in our early studies using qPCR, we revealed an increase in the expression levels of genes associated with cell division, including cell cycle genes and some of meristem regulators, in the spontaneous tumors of radish [5,104]. Thus, data on the upregulation of genes involved in the control of cell division in spontaneous tumors of radish are not unexpected for us.

At the same time, the pathways associated with the response to biotic and abiotic stresses were mostly downregulated in the spontaneous tumors. Among genes involved in these pathways, the most widely represented were genes controlling the biosynthesis of JA and the response to them, as well as genes that regulate the synthesis of specific secondary metabolites that are synthesized when plants are wounded or eaten by insects, for example, glucosinolates and lignin [105,106]. Thus, among downregulated genes, there were radish homologs of the *Arabidopsis* genes that regulate all stages of the JA biosynthesis from the beginning until the end [9], as well as genes that regulate the biosynthesis of glucosinolates [61] and common precursors of glucosinolates and IAA [65,66], and biosynthesis of lignin [58].

These data were also not very unexpected, since the intensification of cell division can lead to tissue juvenilization. The work of age-dependent resistance mechanisms leads to a gain or reinforcement of disease resistance in mature plant organs, whereas juvenile organs with a high level of cell divisions are more susceptible to pathogens [107]. In the adult plant, the activation of age-dependent resistance takes place under the control of genes involved in the regulation of flowering, such as LEAFY [107]. Therefore, the downregulation of genes involved in the flowering control which was revealed in the tumor tissue, may also contribute to the suppression of the age-dependent resistance in the tumor.

The results on changes in the expression of genes that regulate the level of CK and GA and the response to them were quite unexpected for us: based on the data obtained, the balance of these hormones in tumors should sharply shift towards GA. CK and GA are known to antagonistically interact in the control of meristem development, where CK promotes cell division, and GA is responsible for cell growth and differentiation in the periphery [68,69]. Therefore, one should rather expect the activation of CK biosynthesis and suppression of GA biosynthesis in the tumor. Moreover, our previous data indicate the sharp increase of free CK content in the spontaneous tumor of radish [6], and CK were shown to induce tumor formation in radish in vitro [4,108]. It is possible that the downregulation of “CK genes” and an upregulation of “GA genes” revealed by RNA-seq was the result of the work of hypothetical negative feedbacks that reduce the hyperactivation of CK biosynthesis in the radish tumor.

Finally, in the spontaneous tumors, the downregulation of genes that are involved in the control of photomorphogenesis and flowering was revealed. These data were rather unexpected and we can explain the expression of flowering-associated genes in the radish root only by suggesting the broader functions of these genes, e.g., in the radish storage root development. On the other hand, it was reported that individual circadian clock regulators can also participate in providing the plant stress response and affecting SA and JA signaling [109,110]. Therefore, these photomorphogenesis- and flowering-associated genes may be downregulated in the tumor together with other stress-related genes.

Thus, for the first time, data were obtained, which reflect changes in the expression of various gene families in spontaneous tumors in radish lines. A further study of the functions of revealed up- and downregulated genes will help elucidate the mechanisms of plant tumors development.

## 4. Materials and Methods

### 4.1. Plant Material

Line 19 from the genetic collection of *R. sativus* was used in this study. This line is approximately the 40th inbred generation and originated from Saxa cultivar (European group of radish varieties). Line 19 is characterized by a spontaneous tumor formation trait: at the flowering stage, nearly 100% of the plants of line 19 form tumors on the root and lower part of the stem [3].

For RNA sequencing of spontaneous tumors and lateral roots, radish plants of tumor-producing line 19 were grown in field conditions until the flowering stage (about 70 days). Plant material (lateral roots and spontaneous tumors) was harvested on the 15th day from the beginning of flowering, when the tumors became large and clearly visible. To harvest tumor and lateral root samples, plants were removed from the soil and rinsed with a large amount of water. Tumors and root samples were harvested by dissection with a scalpel and immediately preserved in the RNA-later solution (Sigma-Aldrich, Darmstadt, Germany) for further RNA extraction. For both lateral root and tumor samples, three biological replicates, each including material from three individual plants, were used.

### 4.2. RNA Isolation, Library Preparation, and Sequencing

Total RNA from radish plants and seedlings was isolated according to the modified phenol-chloroform method [111]. The cDNA was synthesized with the Mint-2 kit (Evrogen, Moscow, Russia). The NEBNext^®^ Ultra™ DNA Library Prep Kit for Illumina (New England Biolabs, Hitchin, UK) was used to prepare sequencing libraries. Double barcoding was performed using the NEBNext^®^ Ultra™ DNA Index Prep Kit for Illumina and the NEBNext^®^ Multiplex Oligos^®^ Illumina^®^ (Dual Index Primers Set 1). Libraries were sequenced on the Illumina HiSeq2500 sequencer.

### 4.3. Bioinformatic Processing of Sequencing Results

Fastq reads were filtered with bbduk utility from the bbtools suite (v. 38.44) (URL: https://jgi.doe.gov/data-and-tools/bbtools/, accessed on 5 March 2020). To decontaminate reads, we used sequences of mitochondrial, plastid, and ribosomal DNA of radish. Quality filtered and decontaminated reads were additionally trimmed with cutadapt (v. 2.10) and Trimmomatic (v. 0.39) with “ILLUMINACLIP:{ADAPTERS}:2:30:10 LEADING:3 TRAILING:3 SLIDINGWINDOW:4:15 MINLEN:36” options [112]. The quality control of reads was performed with fastqc (v. 0.11.8) (URL: http://www.bioinformatics.babraham.ac.uk/projects/fastqc/, accessed on 20 March 2020). Filtered reads were quantified with kallisto (v. 0.46.1) [113] using RNA sequences from the representative genome assembly of *R. sativus* (NCBI assembly ID GCF_000801105.1). The differential expression between tumor and non-tumor samples was analyzed with the DESeq2 package (v. 1.26.0) [114] for R (v. 3.6.2). Genes with s-value < 0.05 and |logFoldChange| > 1 were considered differentially expressed. For functional annotation and subsequent enrichment analysis of transcripts, genes were compared with *Arabidopsis thaliana* cDNA sequences from the Araport v. 11 [115] base using blast+ (v. 2.6.0) [116]. The R packages ClusterProfiler v. 3.14.0 [117] and DOSE 3.12.0 [118] were applied to perform the GO, KEGG, and gene set enrichment analyses. Gene lists from plantGSEA [119] were used as a reference. FDR < 0.05 was selected as the cut-off value for the functional and pathway enrichment analysis of differentially expressed genes. The code for all the analysis steps of read data is available at https://github.com/castrofiber/radish2_RNAseq (accessed on 10 March 2021). SRA data can be downloaded from http://www.ncbi.nlm.nih.gov/bioproject/686790 (accessed on 1 March 2021).

### 4.4. The qPCR

The data on the differential expression obtained by RNA-seq were verified by the qPCR of selected genes. For qPCR, total RNA was extracted by the Purezol reagent (Bio-Rad, Hercules, CA, USA), purified with chloroform, and precipitated with isopropanol. The RNA pellet was washed three times with 80% ethanol, dried under air flow in a laminar box, and dissolved in sterile deionized water. The DNase treatment was done using the Rapid Out DNA Removal Kit (Thermo Fisher Scientific, Waltham, MA, USA). The RNA concentration was measured by the NanoDrop 2000 UV spectrophotometer (Thermo Fisher Scientific) at 260 nm. For reverse transcription, 500 ng of RNA were used in all the samples. The RNA reverse transcription was performed using the Revert Aid Reverse Transcriptase kit (Thermo Fisher Scientific). To check the DNase treatment efficacy, the qRT-PCR analysis of control samples without the reverse transcriptase was performed. The qRT-PCR experiments were done on a CFX-96 real-time PCR detection system with the C1000 thermal cycler (Bio-Rad), and Eva Green intercalating dye (Syntol, Moscow, Russia) was used for detection. Primers for qRT-PCR (Table S1) were designed to amplify 140–200 bp fragments and were synthesized by Evrogen. The specificity of PCR amplification was confirmed based on the melting curve (55–95 °C). All the reactions were performed in technical triplicate and averaged. Cycle threshold values were obtained with the accompanying CFX manager software, and data were analyzed by the 2^−ΔΔCt^ method [120]. The relative expression was normalized against constitutively expressed *R. sativus* ubiquitin (*RsUBQ11*) and glyceraldehyde-3-phosphate dehydrogenase (*RsGAPDH*) genes [121]. Experiments were repeated three times with independent biological samples, whose results were then averaged.

## Data Availability

The scripts, programs, and pipeline used for the RNA-seq analysis are available at https://github.com/castrofiber/radish2_RNAseq (accessed on 22 April 2021). Raw reads are available at https://www.ncbi.nlm.nih.gov/bioproject/686790 (accessed on 22 April 2021).

## Supplementary Materials

The following materials are available online at https://www.mdpi.com/article/10.3390/plants10050919/s1. Table S1: List of primers used for qPCR, Table S2: Results of differential expression analysis of tumor samples in comparison with lateral roots.

## Abbreviations

ABA—abscisic acid, CK—cytokinin, DEG—differentially expressed gene, GA—gibberellic acid, IAA—indole-3-acetic acid, JA—jasmonic acid, SA—salycilic acid, TF—transcription factor.
