# Supplementary material for: Transcriptomic Analysis of Radish (Raphanus sativus L.) Spontaneous Tumor
Source: Plants (Basel). 2021 May 3;10(5):919. doi: 10.3390/plants10050919 (PMC8147785; doi:10.3390/plants10050919)
Supplement: Supplementary file 1 [file plants-10-00919-s001.zip › Table S1.pdf]

Table S1. List of primers used for qPCR.

Gene name	NCBI identifier	Amplicon size, bp	Orientation	Primer sequence, 5’-3’	GC, %	Tm, ºC
Target genes						
RsCYCA1;1	*XM_018592281.1*	158	Forward	ATGTCGAACATTCATCATCAGAATCGC	40.7	61.1
			Reverse	CGTGAACCCTTCTTTTGATTTGTGATG	40.7	60.7
RsCYCB1;2	*XM_018617721.1*	148	Forward	ATGGCGACGAGAACCAACATG	52.4	57.1
			Reverse	CGGGAACGGAAACAAGGTTG	55	56.7
RsDEL1	*XM_018603289.1*	163	Forward	TGGGATCAAGAGGTTATGAGTTAGGT	51.3	55.5
			Reverse	CTCACGACCTTCACTCTCTCGG	51.3	57.6
RsEXPA3	*XM_018581553.1*	191	Forward	ATGGCGGCGACTGCGTTTAGAA	54.5	62.4
			Reverse	TATAAGTTCCCGTACCCACACGCACC	53.8	62.7
RsARR4	*XM_018624193.1*	169	Forward	GGAACCTCCGCCGTTAGATTTAGA	52.3	58.7
			Reverse	TTTCTCGTTGTCTAGCCCTAGAAGC	52.6	56.5
RsARR5	*XM_018581651.1*	157	Forward	ATGTCGAACATTCATCATCAGAATCG	38.5	58.1
			Reverse	GTGAACCCTTCTTTTGATTTGTGATGT	37.0	57.7
RsGA20OX3	*XM_018633395.1*	159	Forward	GCGAAAGGGAAAGGAGGTCAT	49.2	56.1
			Reverse	CGGATCTATAATGCTTCTGCGTGA	50.6	57.3
RsCLE46	*XM_018604748.1*	167	Forward	TCCATCTTTTATGCCCATCC	44.6	52.5
			Reverse	GGTCAAAGTCAAGCCTGAGCC	51.2	55.3
RsLBD25	*XM_018597567.1*	177	Forward	CGAACTACACAAACTCACGGTGC	51.9	56.2
			Reverse	TCCGTATGGAGTTGAGAGGTGAGA	49.2	56.2
RsLBD38	*XM_018581284.1*	167	Forward	ACGCCACCGTATTCGTCGCTA	51.2	59.1
			Reverse	TGGCATTGAGTAATCCGTCA	52.0	60.4
RsWRKY9	*XM_018604382.1*	148	Forward	CATGTGAAAGAGTAGAATAAGAGGTTGAG	52.2	54.7
			Reverse	CTTAGCATGTTCTCCAAGGAACG	50.2	54.8
RsENO2	*XM_018580867.1*	187	Forward	GGTAGGTTCAAGGGCGAGAGAGTT	54.2	58.0
			Reverse	CCGATGATGTTGTTCACATTGC	47.9	55.2
RsDPB	*XM_018587639.1*	157	Forward	ATGACAACAACAACAACAAGTGGGTC	51.3	57.9
			Reverse	CGCCGCTAGTGGATACCGATT	51.2	57.8
RsAOC3	*XM_018598854.1*	155	Forward	GTTCCTCGCCGTCACTTGTGG	53.1	59.1
			Reverse	GTAACCGCCGTCCCAGTAAGC	53.1	57.4
RsIMS3	*XM_018608445.1*	178	Forward	CGTCACTTCTGACATCTCCCACAAT	52.6	58.1
			Reverse	GTGACGATGTTGTTGTTGTTTTCG	54.6	57.8
RsIPT5	*XM_018587590.1*	199	Forward	ACACTCACGACGATTTCACG	46.6	51.7
			Reverse	ACGACGAGGGATCGAATATG	46.7	51.5
RsIPT7	*XM_018633594.1*	197	Forward	ATGAAGTTCTCAATCTCAGCAATGAAGC	51.9	59.3
			Reverse	GAGCAAGTGGTGAGGCACGC	52.8	58.0
RsYUC8	*XM_018594055.1*	145	Forward	ATGGAGAGTATGTTACGTTTGATGGAT	40.7	56.6
			Reverse	AAGGAACGTTTTGCTCCCGG	50.0	55.7
RsBFT	*XM_018581284.1*	169	Forward	GTGGGAAGAGTGATAGGAGATGTCAT	52.9	55.7
			Reverse	TGAAGAATGAACGGAGATCATGG	48.4	55.5
RsSOC1	*XM_018623442.1*	192	Forward	GAGGGGAAAAACTCAGATGAAGAGG	52.6	57.6
			Reverse	GGTATCTTGTATATTGGAGATGGCGA	51.3	56.6
RsBELL4	*XM_018603518.1*	144	Forward	GATTCCACAGACCATCACCCTCC	53.7	51.7
			Reverse	ACGACGAGGGATCGAATATG	46.7	51.5
RsTCP2	*XM_018608972.1*	144	Forward	GACGACAACCTCCACCGCC	52.4	57.4
			Reverse	CGTAACCGATGCCTCTAACCTCAGAA	53.4	58.4
RsRL3	*XM_018614337.1*	165	Forward	TGGCTTCCATCTCCATGAGTTCTA	50.6	56.7
			Reverse	CTCGTAGTGTCTCCTTACTTCTTCAGC	54.6	55.5
RsNAC90	*XM_018588875.1*	164	Forward	GCTTTTGATAGACGCCCATTGG	49.7	57.4
			Reverse	ACCATCTACAATCTCTCCATTCATAGAC	51.9	54.4
Reference genes						
RsUBQ11	*XM_018620761.1*	197	Forward	GCCGACTACAACATCCATAAGGAG	50.0	55.9
			Reverse	AAAGATCAACCTCTGCTGGTCCG	52.2	58.3
RsGAPDH	*XM_018627555.1*	199	Forward	TCTCTTCGGTGAGAAGCCAGTCA	51.9	57.8
			Reverse	TCAAGTGAGCAGCAGCCTTGTC	51.6	57.0
